# Macrophage polarization state affects lipid composition and the channeling of exogenous fatty acids into endogenous lipid pools

**DOI:** 10.1016/j.jbc.2021.101341

**Published:** 2021-10-23

**Authors:** Pooranee K. Morgan, Kevin Huynh, Gerard Pernes, Paula M. Miotto, Natalie A. Mellett, Corey Giles, Peter J. Meikle, Andrew J. Murphy, Graeme I. Lancaster

**Affiliations:** 1 Baker Heart and Diabetes Institute, Melbourne, Australia; 2 School of Life Sciences, La Trobe University, Melbourne, Australia; 3 Department of Anatomy and Physiology, School of Biomedical Sciences, University of Melboure, Melbourne, Australia; 4 Department of Immunology, Monash University, Melbourne, Australia

**Keywords:** macrophage, fatty acid metabolism, phospholipid metabolism, sphingolipid, cell metabolism, AA, arachidonic acid, ATM, adipose-tissue-resident macrophage, BMDM, bone-marrow-derived macrophage, CE, cholesterol ester, DG, diacylglycerol, DHA, docosahexaenoic acid, FACS, fluorescence-activated cell sorting, FBS, fetal bovine serum, GPL, glycerophospholipid, PC, phosphatidylcholine, PCA, principal component analysis, PE, phosphatidylethanolamine, PG, phosphatidylglycerol, PGE_2_, prostaglandin E2, PI, phosphatidylinositol, PUFA, polyunsaturated fatty acid, TG, triacylglycerol, TLR, toll-like receptor

## Abstract

Adipose-tissue-resident macrophages (ATMs) maintain metabolic homeostasis but also contribute to obesity-induced adipose tissue inflammation and metabolic dysfunction. Central to these contrasting effects of ATMs on metabolic homeostasis is the interaction of macrophages with fatty acids. Fatty acid levels are increased within adipose tissue in various pathological and physiological conditions, but appear to initiate inflammatory responses only upon interaction with particular macrophage subsets within obese adipose tissue. The molecular basis underlying these divergent outcomes is likely due to phenotypic differences between ATM subsets, although how macrophage polarization state influences the metabolism of exogenous fatty acids is relatively unknown. Herein, using stable isotope-labeled and nonlabeled fatty acids in combination with mass spectrometry lipidomics, we show marked differences in the utilization of exogenous fatty acids within inflammatory macrophages (M1 macrophages) and macrophages involved in tissue homeostasis (M2 macrophages). Specifically, the accumulation of exogenous fatty acids within triacylglycerols and cholesterol esters is significantly higher in M1 macrophages, while there is an increased enrichment of exogenous fatty acids within glycerophospholipids, ether lipids, and sphingolipids in M2 macrophages. Finally, we show that functionally distinct ATM populations *in vivo* have distinct lipid compositions. Collectively, this study identifies new aspects of the metabolic reprogramming that occur in distinct macrophage polarization states. The channeling of exogenous fatty acids into particular lipid synthetic pathways may contribute to the sensitivity/resistance of macrophage subsets to the inflammatory effects of increased environmental fatty acid levels.

Macrophages are cells of the innate immune system with critical roles in the elimination of infectious microorganisms, the resolution of tissue damage, and the maintenance of tissue and organismal homeostasis (1). Macrophages are also implicated in the etiology of numerous diseases, particularly chronic conditions where nonresolving inflammation contributes to disease pathogenesis (2, 3). The involvement of macrophages in such diverse aspects of homeostatic and inflammatory biology is principally due to their plasticity, with developmental and environmental signals reprogramming macrophages, leading to the acquisition of specific phenotypic states (4).

One of the many tissue-specific signals that macrophages are exposed to is altered nutrient levels. This is exemplified in the adipose tissue and liver, where resident macrophages are exposed to marked fluctuations in fatty acid levels that result from physiological challenges such as fasting (5, 6) and cold exposure (7) or pathological conditions such as obesity (8, 9). The metabolism of extracellular lipids by adipose- and liver-resident macrophages is associated with two distinct outcomes. Firstly, by buffering fluctuations in tissue lipid levels, adipose tissue- and liver-resident macrophages help to maintain metabolic homeostasis (5, 10, 11). Moreover, it was recently demonstrated that adipose-tissue-resident macrophages (ATMs) facilitate lipid storage within white adipocytes, providing an additional means by which ATMs contribute to metabolic homeostasis (12). Secondly, increased tissue lipid levels can lead to triacylglycerol (TG) accumulation and inflammation within macrophages, thereby contributing to the development of metabolic dysfunction (13, 14).

Macrophage phenotypes have conventionally been classified as belonging to either M1 or M2 polarization states (15, 16). M1 macrophages express high levels of proinflammatory mediators (iNOS-NO, cycloxygenase-2 (Cox2)-prostaglandin E2 (PGE_2_), proinflammatory cytokines) and are antimicrobial (16, 17). The long-term presence of M1 macrophages in tissues is linked to the development of numerous chronic diseases, *e.g.*, insulin resistance, type 2 diabetes, fatty liver disease (3, 18, 19). M2 macrophages express high levels of Arginase1-ornithine, anti-inflammatory cytokines, and scavenger receptors and mediate tissue repair and homeostasis (16, 17, 20). These functional differences between M1 and M2 macrophages are supported by a substantial rewiring of cellular metabolism (21, 22). Accordingly, M1 macrophages have reduced mitochondrial oxidative capacity, increased rates of glycolysis and *de novo* fatty acid synthesis, and contain TG-rich lipid droplets (23, 24, 25). M2 macrophages are characterized by a reduced glycolytic rate, but increased rates of fatty acid oxidation, with lipid droplets being largely absent from M2 macrophages (23, 25). Importantly, many of these classical features of M1 and M2 macrophages are shared with adipose tissue and liver-resident macrophages *in vivo*. Thus, ATM subsets associated with obesity and metabolic dysfunction are M1-like, displaying increased TG accumulation and being inflammatory (13, 14). Conversely, the macrophage subsets in the liver and adipose tissue that are linked to the maintenance of metabolic homeostasis have an M2-like transcriptional signature associated with tissue homeostasis, display increased lipid catabolic pathways, and are not inflammatory (5, 10, 11, 13). Why elevated fatty acid levels—that are characteristic of both obesity and numerous physiological stimuli—only initiate inflammatory responses in particular macrophage subsets is unclear, but may be related to the underlying macrophage phenotypic state. In the present study we used a combination of targeted lipidomics and stable-isotope labeling to examine how macrophage polarization state influences the metabolic fate of extracellular-derived fatty acids.

## Results

### M1 macrophages have increased TG levels and a shift in compositional profile toward polyunsaturated fatty acid–containing TGs

**Figure 1:**
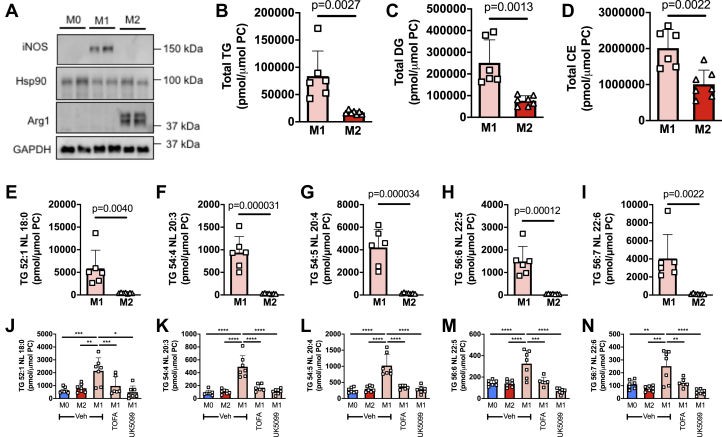
**Basal neutral lipids levels are higher in M1 relative to M2 macrophages.***A*, Western blot analysis demonstrating the polarization of BMDM to the M1 and M2 states following treatment with either LPS+IFNγ (M1) or IL-4 (M2). Nonpolarized (M0) BMDM are shown for comparison. Two biological replicates are shown for each condition. Levels of total TG (*B*), total DG (*C*), total CE (*D*), and multiple individual TG species (*E–I*) in M1 and M2 BMDM. Levels of individual TG species in M0, M2, and in M1 macrophages treated with or without TOFA (acetyl-CoA carboxylase inhibitor) or UK5099 (mitochondrial pyruvate carrier inhibitor) (*J*–*N*). In (*B*–*I*), data was analyzed by unpaired *t* test with exact *p* values indicated. In (*J*–*N*), data was analyzed by one-way ANOVA. ∗, ∗∗, ∗∗∗, and ∗∗∗∗ indicate significant differences between pairwise comparisons of the indicated groups at the following significance level: *p* < 0.05, *p* < 0.01, *p* < 0.001, and *p* < 0.00001, respectively. Data are shown as mean ± S.D. (error bars) as well as with individual data points from each biological replicate.

To determine how macrophage phenotypic state influences the metabolism of exogenous fatty acids, we firstly generated bone-marrow-derived M0 macrophages (unpolarized), which were subsequently polarized to the M1 (LPS + IFNy for 24 h) or M2 (IL-4 for 24 h) state (Fig. 1*A*) and then performed mass-spectrometry-based targeted lipidomic profiling. In the absence of exogenously supplied fatty acids, with the exception of those FAs and other lipids present in serum, we observed that M1 macrophages have substantially higher levels of TGs, diacylglycerols (DGs), and cholesterol esters (CEs) (Fig. 1, *B*–*D*), consistent with the results of previous studies (26, 27, 28). Interestingly, in addition to a difference in the abundance of TG between M1 and M2 macrophages, we observed a significant compositional change in the TG pool (Fig. S1). Specifically, the profile of TGs in M2 macrophages was more saturated, with >60% of TGs being fully saturated (*i.e.*, containing no double bonds in any of the fatty acyl chains). In contrast, M1 macrophages had a higher proportion of TGs containing unsaturated fatty acids, in particular TGs containing polyunsaturated fatty acids (PUFAs), *i.e.*, TGs containing six to ten double bonds within their constituent fatty acyl chains. These differences are the result of a substantial increase in TG levels in M1 macrophages, which, although occurring across all TG species, are most marked in PUFA-containing TGs. Figure 1, *E*–*I* provide examples of the changes in TGs containing fatty acids of varying saturation status. Previous work would indicate that the increase in TG levels in M1 macrophages is likely due to increased fatty acid supply *via de novo* synthesis as well as an increase in the expression of multiple enzymes in the TG synthesis pathway (26, 28). Indeed, blocking *de novo* fatty acid synthesis during M1 macrophage polarization by inhibiting either acetyl-CoA carboxylase (TOFA) or the mitochondrial pyruvate transporter (UK5099) completely prevented the increase in TG species (Fig. 1, *J*–*N*). Of note, PUFAs cannot be synthesized *de novo*; therefore, in the case of increasing PUFA-containing TG species, the PUFA likely derives from serum *via* extracellular uptake or potentially from phospholipid remodeling. Indeed, we observed lower levels of numerous PUFA-containing phospholipid species in M1 compared with M2 macrophages (compare the BSA groups in Fig. S3, *C*, *D*, *F*, and *K*), consistent with the possibility that the breakdown of PUFA-containing glycerophospholipids (GPLs) may supply PUFAs that are subsequently used in TG synthesis.

### M1 macrophages have increased TG and CE levels following treatment with exogenous fatty acids

**Figure 2:**
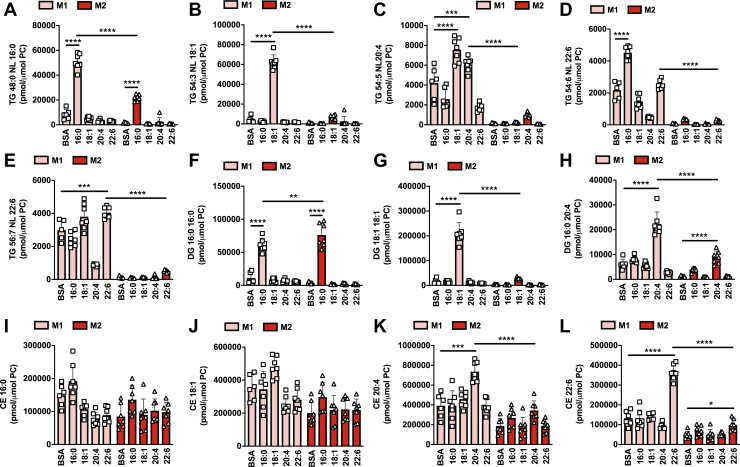
**Exogenous fatty acids increase neutral lipid levels to a greater extent in M1 relative to M2 macrophages.** M1 and M2 BMDMs were treated individually for 4 h with 200 μM palmitic acid (16:0), oleic acid (18:1), arachidonic acid (20:4), docosahexaenoic acid (22:6), or vehicle control (BSA). Samples were analyzed by MS lipidomics and the levels of individual TG species (*A*–*E*), DG species (*F*–*H*), and CE species (*I*–*L*) were determined. Two-way ANOVA with Tukey’s HSD test was used to determine statistically significant differences. ∗, ∗∗, ∗∗∗, and ∗∗∗∗ indicate significant differences between pairwise comparisons of the indicated groups at the following significance level: *p* < 0.05, *p* < 0.01, *p* < 0.001, and *p* < 0.00001, respectively. Data are shown as mean ± S.D. (error bars) as well as with individual data points from each biological replicate.

Within the adipose tissue, adipocytes are constantly undergoing lipolysis, releasing fatty acids that can be sensed by resident macrophages. We aimed to explore how macrophage polarization state (M1 *versus* M2) influenced the metabolism of the major fatty acid species that are released following adipocyte lipolysis. Thus, we exposed M1 and M2 macrophages to exogenously added palmitic acid (Pal; 16:0), oleic acid (Ol; 18:1), arachidonic acid (AA; 20:4), and docosahexaenoic acid (DHA; 22:6). Mass-spectrometry-based targeted lipidomic profiling was used to determine the endogenous lipid species that were altered following treatment with exogenous fatty acids. Macrophage polarization state had a marked impact on the fate of exogenous fatty acids. Strikingly, the levels of numerous TG (Fig. 2, *A*–*E*), DG (Fig. 2, *F*–*H*), and CE species (Fig. 2, *I*–*L*) were higher in M1 macrophages following Pal, Ol, and AA treatment. Notably, the fate of exogenous DHA was distinct from Pal and Ol. Specifically, while Pal and Ol markedly increased the levels of TG species containing a 16:0 or 18:1 fatty acyl chain (Fig. 2, *A* and *B*), DHA treatment had relatively minor effects on the levels of DHA-containing TG species (Fig. 2, *D* and *E*). In contrast, while exogenous Pal and Ol led to only minor increases in 16:0- and 18:1-containing CEs (Fig. 2, *I* and *J*), 22:6-containing CEs were markedly increased following DHA treatment (Fig. 2*L*). Exogenous AA increased both 20:4-containing TG and CE species (Fig. 2, *C* and *K*). These results demonstrate that in addition to having higher basal levels of neutral lipids, M1 macrophages accumulate significantly more TG and CE than M2 macrophages following treatment with exogenous fatty acids. They also demonstrate that the fate of exogenous fatty acids within M1 macrophages is dependent upon the saturation status/acyl chain length, with saturated and monounsaturated fatty acids (16:0 and 18:1) preferentially accumulating within TGs and PUFA preferentially accumulating within CEs.

### The accumulation of exogenous fatty acids within glycerophospholipids is higher in M2 macrophages

TG and CE accumulation following exogenous fatty acid treatment is considerably lower in M2 macrophages (Fig. 2). While the well-described higher fatty acid oxidation capacity of M2 macrophages (21, 23) likely contributes to this effect, we hypothesized that M2 macrophages may possess additional metabolic alterations that contribute to the reduced accumulation of TGs and CEs. Firstly, to determine if altered rates of fatty acid uptake may contribute to the differences described above, we assessed the relative fatty acid uptake capacities of M1 and M2 macrophages. To do this, we used a click-chemistry-based approach in which macrophages are treated with functionalized fatty acids (*i.e.*, fatty acids in which the terminal methyl group is replaced by an alkyne) followed by conjugation *via* copper-catalyzed azide-alkyne cycloaddition to an azide-Alexa488 conjugate to enable the assessment of fatty acid uptake by flow cytometry. M2 macrophages had a significantly higher uptake of alkyne-oleate and alkyne-palmitate compared with M1 macrophages (Fig. S2), demonstrating that reduced fatty acid uptake is not responsible for the lower accumulation of TG, DG, and CE observed in M2 macrophages following treatment with exogenous fatty acids.

We hypothesized that exogenous fatty acids are incorporated into alternate lipid biosynthetic pathways in M2 macrophages, thereby reducing their esterification within TGs and CEs. GPLs are the major constituents of cell membranes, with important structural and biochemical roles. GPLs consist of a glycerol backbone with ester-linked fatty acyl chains and a variable (*e.g.*, choline, serine, ethanolamine) phosphate ester head group. Treatment with exogenous fatty acids increased the levels of multiple GPL species from different classes, including phosphatidylcholine (PC) (Fig. S3, *A*–*H*), phosphatidylethanolamine (PE) (Fig. S3, *I*–*N*), phosphatidylinositol (PI) (Fig. S3, *O*–*R*), phosphatidylglycerol (PG) (Fig. S3*S*), and phosphatidylserine lipid species (Fig. S3*T*). As expected, the GPL species altered were generally those containing acyl chains that corresponded to the specific exogenously added fatty acid; *e.g.*, PE 18:1/18:1 was increased only following exposure to oleic acid. Treatment with exogenous fatty acids increased the abundance of multiple PC and PE species in both M1 and M2 macrophages (Fig. S3, *A*–*N*). Interestingly, while treatment with Pal and Ol resulted in similar increases in PE and PC species containing 16:0 and 18:1 acyl chains, the increase in PC and PE species containing 20:4 and 22:6 acyl chains following treatment with exogenous AA and DHA was significantly greater in M2 relative to M1 macrophages (Fig. S3, *A*–*N*). Similarly, the increase in numerous PI species was significantly greater in M2 macrophages (Fig. S3, *O*–*R*).

Ether lipids are a type of GPL that contain a fatty alcohol linked either *via* an ether or vinyl-ether bond at the Sn1 position of glycerol. Like their diacyl counterparts, ether GPLs are abundant membrane lipids, but their altered chemical linkage endows ether lipids, and in particular vinyl-ether lipids (plasmalogens), with unique biophysical and biochemical properties (29). Treatment with exogenous fatty acids increased the levels of ether PCs (Fig. S4, *A*–*E*; PC(O)) and both vinyl-ether PCs (Fig. S4, *F*–*I*; PC(P)) and PEs (Fig. S4, *J*–*P*; PE(P)). Where exogenous fatty acids increased the level of a specific ether/vinyl-ether GPL, these increases were generally significantly greater in M2 macrophages (Fig. S4).

**Figure 3:**
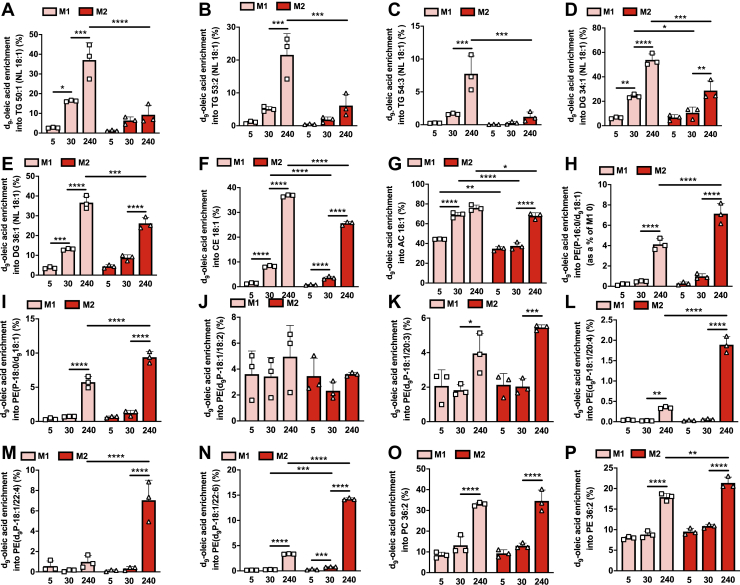
**Analysis of deuterated (d**_**9**_**)-oleic acid enrichment within the lipidome of M1 and M2 macrophages reveals marked rewiring of exogenous fatty acid metabolism.** M1 and M2 BMDMs were treated with d_9_-oleic acid for the indicated durations and enrichment within individual TG (*A*–*C*), DG (*D* and *E*), CE (*F*), acylcarnitine (*G*), vinyl-ether glycerophospholipid (*H*–*N*), and glycerophospholipid (*O* and *P*) was determined by MS lipidomics. Two-way ANOVA with Tukey’s HSD test was used to determine statistically significant differences. ∗, ∗∗, ∗∗∗, and ∗∗∗∗ indicate significant differences between pairwise comparisons of the indicated groups at the following significance level: *p* < 0.05, *p* < 0.01, *p* < 0.001, and *p* < 0.00001, respectively. Data are shown as mean ± S.D. (error bars) as well as with individual data points from each biological replicate.

A limitation to measuring the abundance of intracellular lipid species using conventional MS-lipidomics with the goal of determining the fate of exogenous fatty acids is that the levels observed reflect both endogenous and exogenous lipid sources. Therefore, to gain greater insight specifically into the fate of exogenous fatty acids within M1 and M2 macrophages, we utilized stable-isotope labeled fatty acid tracers. To gain insight into the rate of exogenous fatty acid incorporation into various endogenous lipid pools, we treated M1 and M2 macrophages for 5, 30, and 240 min with deuterated oleate (d_9_-oleate). Consistent with the data presented above, d_9_-oleate was enriched within TGs, DGs, and CEs at substantially greater levels in M1 relative to M2 macrophages (Fig. 3, *A*–*F*). Of note, d_9_-oleate enrichment within TGs, DGs, and CEs in M1 macrophages was observable after as little as 5 min of treatment and was markedly increased after only 30 min. This contrasts with M2 macrophages in which detectable increases in TGs, DGs, and CEs were only apparent after 30 and 240 min. A marked d_9_-oleate enrichment within 18:1 acylcarnitine was also observed in M1 macrophages, which was significantly lower in M2 macrophages (Fig. 3*G*). The enrichment of d_9_-oleate within ether and vinyl-ether GPLs was significantly higher in M2 macrophages (Fig. 3, *H*–*N*). Owing to the specific fragmentation of PE plasmalogens in our lipidomics profiling conditions (30), we were able to distinguish d_9_-oleate incorporation to the acyl and vinyl-ether chains. We observed d_9_-oleate incorporation into PE plasmalogen species containing 18:1 at either the Sn2 acyl (Fig. 3, *H* and *I*) or Sn1 alkenyl positions (Fig. 3, *J*–*N*), indicating that exogenous fatty acids can be both reduced to fatty alcohols to serve as substrates for the peroxisomal synthesis of 1-alkyl-DHAP as well as serve as substrates for acyl/alkyl-G3P-acyltransferase, which catalyzes the addition of an acyl chain to the Sn2 position of 1-alkyl-G3P (the product of 1-alkyl-DHAP metabolism) (29). Interestingly, d_9_-oleate enrichment was markedly higher in PE plasmalogen species that contained a PUFA at the Sn2 position (Fig. 3, *L*–*N*). As expected, d_9_-oleate was also enriched within major 18:1-containing PC and PE GPLs (Fig. 3, *O* and *P*), which tended to be higher in M2 macrophages. The results from this time course analysis were confirmed in an independent experiment performed in M1 and M2 macrophages treated with d_9_-oleate for 4 h (Fig. S5, *A*–*P*).

**Figure 4:**
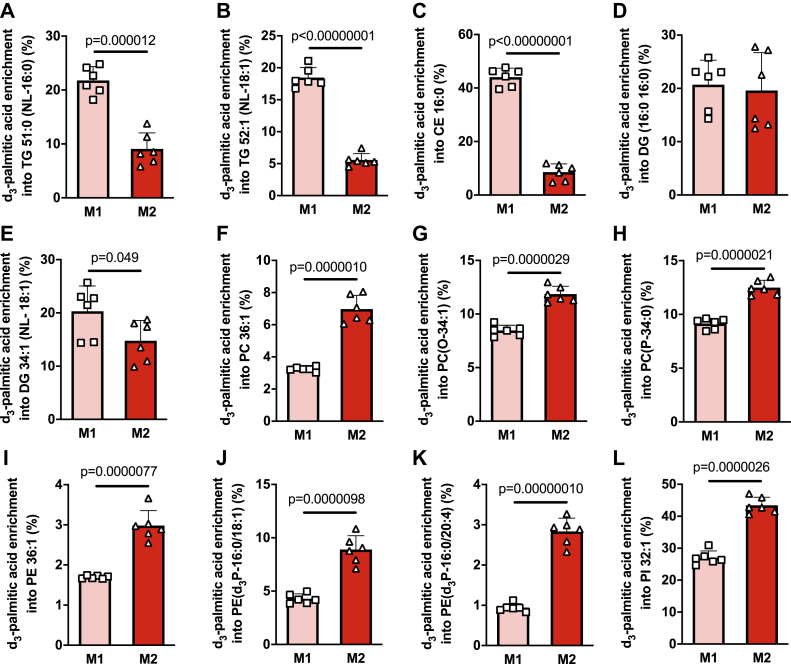
**Analysis of d**_**3**_**-palmitic acid enrichment within the lipidome of M1 and M2 macrophages reveals marked rewiring of exogenous fatty acid metabolism.** M1 and M2 BMDMs were treated with d_3_-palmitic acid for 4 h and enrichment within individual TG (*A* and *B*), CE (*C*), DG (*D* and *E*), glycerophospholipid (*F* and *L*), ether glycerophospholipid (*G*), and vinyl-ether glycerophospholipid (*H*–*K*) was determined by MS lipidomics. Data was analyzed by unpaired *t* test with exact *p* values indicated. Data are shown as mean ± S.D. (error bars) as well as with individual data points from each biological replicate.

Next, we assessed the enrichment of deuterated palmitate (d_3_-palmitate) within specific lipid species in M1 and M2 macrophages. As expected, d_3_-palmitate was enriched in lipid species from multiple lipid classes. Consistent with the results described above using either d_9_-oleate or nonlabeled fatty acids, the enrichment of d_3_-palmitate within TG and CE species was significantly greater in M1 macrophages (Fig. 4, *A*–*C*). Enrichment of d_3_-palmitate in DGs was unaffected by macrophage polarization state (Fig. 4, *D* and *E*). In contrast, the enrichment of d_3_-palmitate within various diacyl, ether, and vinyl-ether GPLs was significantly greater in M2 macrophages (Fig. 4, *F*–*L*). Collectively, these results demonstrate that M2 macrophages have a propensity to channel exogenous fatty acids into glycerophospholipid synthetic pathways. This effect likely contributes to the reduced accumulation of TGs and CEs observed in M2 macrophages.

### M2 macrophages have an increased channeling of exogenous fatty acids into sphingolipids

**Figure 5:**
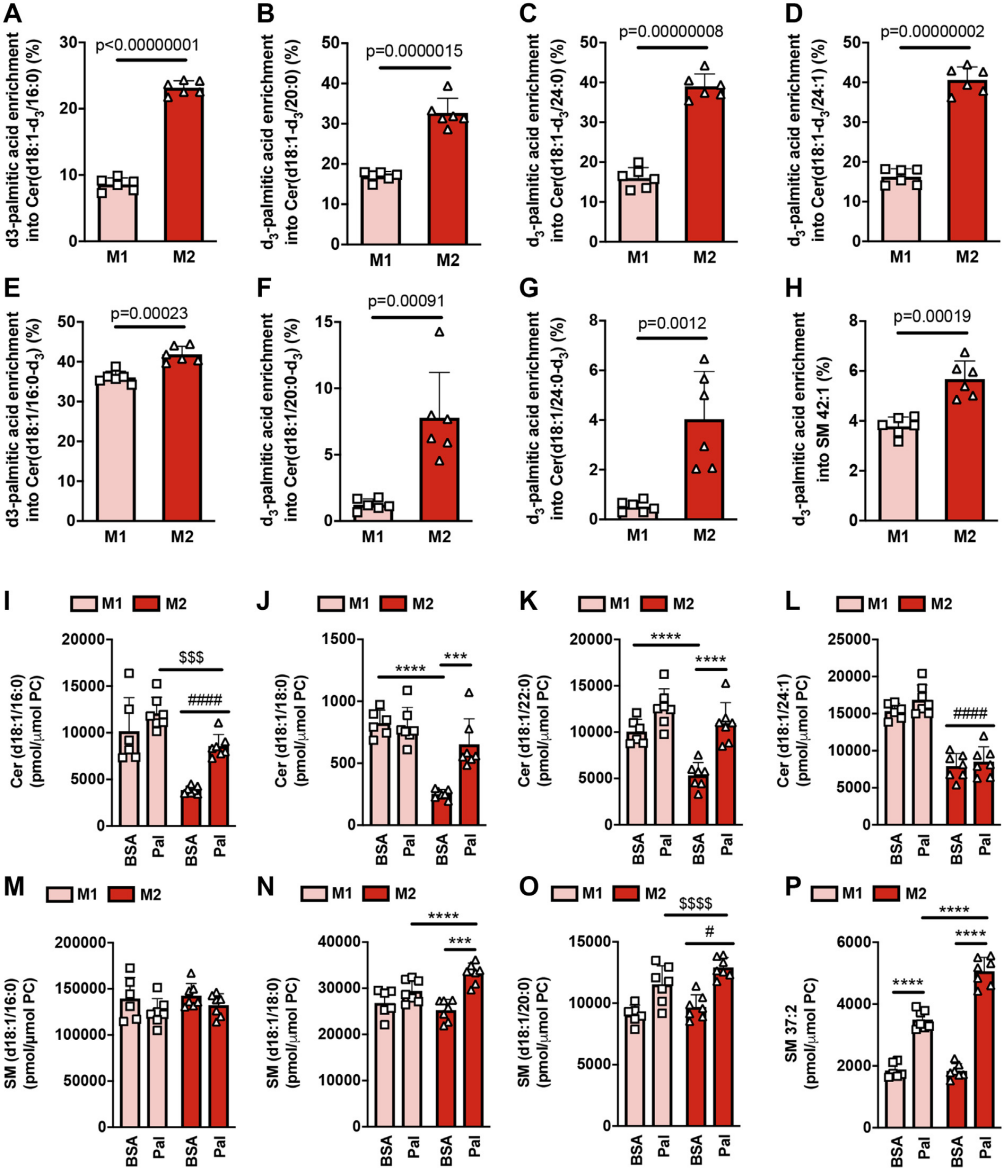
***De novo* sphingolipid synthesis is higher in M2 relative to M1 macrophages.***A*–*H*, M1 and M2 BMDMs were treated with d_3_-palmitic acid for 4 h and enrichment within individual sphingolipid species was determined by MS lipidomics. Data was analyzed by unpaired *t* test with exact *p* values indicated. *I*–*P*, M1 and M2 BMDMs were treated for 4 h with 200 μM palmitic acid (Pal) or vehicle control (BSA) and samples analyzed by MS lipidomics to determine the levels of individual sphingolipid species. Two-way ANOVA with Tukey’s HSD test was used to determine statistically significant differences. ∗∗∗ and ∗∗∗∗ indicate significant differences between pairwise comparisons of the indicated groups at the following significance level: *p* < 0.001 and *p* < 0.00001, respectively. $$$ and $$$$ indicate a significant difference (main effect) between palmitate and BSA-treated BMDM at the following significance level: *p* < 0.0001 and *p* < 0.00001. # and #### indicate a significant difference (main effect) between M1 and M2 BMDM at the following significance level: *p* < 0.05 and *p* < 0.00001. Data are shown as mean ± S.D. (error bars) as well as with individual data points from each biological replicate.

Because palmitate is the preferred substrate in the formation of the sphingoid base that forms the backbone of ceramides and other sphingolipids (31), we were particularly interested in examining how macrophage polarization state affected the incorporation of d_3_-palmitate into ceramide and other sphingolipids. Sphingolipids also contain a second, typically saturated, long-chain fatty acid (16–26 carbons in length) (31). Accordingly, we assessed the enrichment of d_3_-palmitate within both the sphingoid base and the acyl chain of major ceramide species. We observed considerable d_3_-palmitate enrichment within the sphingoid base of multiple d18:1 sphingoid base-containing ceramide species in both M1 and M2 macrophages, indicative of substantial entry of palmitate into the *de novo* ceramide synthesis pathway (Fig. 5, *A*–*D*). Strikingly, d_3_-palmitate enrichment within the d18:1 sphingoid base was markedly higher in M2 macrophages (Fig. 5, *A*–*D*). With regard to the incorporation of d_3_-palmitate within the ceramide acyl chain, a marked increase in Cer(d18:1/16:0-d_3_) was observed in both M1 and M2 macrophages, and this was slightly, although significantly, higher in M2 macrophages (Fig. 5*E*). Furthermore, while very little d_3_-palmitate enrichment within the acyl chains of Cer(d18:1/20:0-d_3_) and Cer(d18:1/24:0-d_3_) was observed in M1 macrophages, significant d_3_-palmitate enrichment into these ceramide species was observed in M2 macrophages (Fig. 5, *F* and *G*), indicating active fatty acid elongation, *e.g.*, from 16:0 to 18:0, 20:0, 22:0, and 24:0. With the exception of SM 42:1, we did not observe d_3_-palmitate enrichment across multiple measured sphingomyelin species (Fig. 5*H*); likely reflecting the relatively short incubation time of macrophages with d3-palmitate (4 h). With regard to SM 42:1, while d_3_-palmitate enrichment was observed in both M1 and M2 macrophages, this was significantly higher in M2 macrophages (Fig. 5*H*). These findings using d_3_-palmitate are consistent with results obtained using non-stable-isotope-labeled palmitate. Specifically, several d18:1 sphingoid base-containing ceramide species were increased following palmitate treatment, effects that were significantly greater in M2 relative to M1 macrophages (Fig. 5*, *I*–L*). Very few sphingomyelin species were increased following palmitate treatment; however, among those sphingomyelin species that were increased following palmitate treatment, these increases were typically significantly greater in M2 macrophages (Fig. 5, *M*–*P*). Collectively, these results demonstrate an increased entry of exogenous palmitate into the *de novo* ceramide synthetic pathway in M2 macrophages, which, similar to the case for increased glycerophospholipid synthesis, may contribute to the reduced accumulation of TGs and CEs observed in M2 macrophages.

## Investigating the lipid profile of CD9^+^ and Ly6C^+^ adipose-tissue-resident macrophages

**Figure 6:**
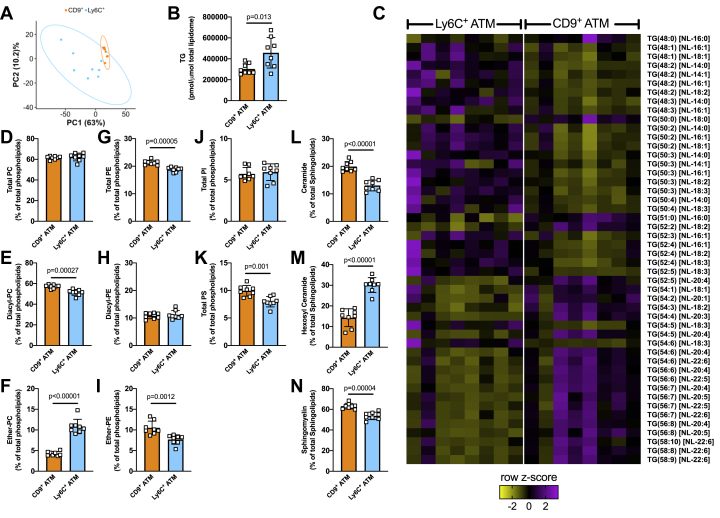
**Analysis of the lipidome of CD9**^**+**^**and Ly6C**^**+**^**adipose-tissue-resident macrophages from high-fat-fed mice.***A*, principal components analysis (PCA) of the global lipidome. *B*, TG levels in CD9^+^ and Ly6C^+^ macrophages. *C*, heat map showing significantly different neutral loss (NL) TG species. Phospholipid—total PC (*D*), diacyl-PC (*E*), ether-PC (*F*), total PE (*G*), diacyl-PE (*H*), ether-PE (*I*), PI (*J*), PS (*K*); and sphingolipid—ceramide (*L*), hexosyl-ceramide (*M*), and sphingomyelin (*N*) levels in CD9^+^ and Ly6C^+^ macrophages. In (*B* and *D*–*N*), data was analyzed by unpaired *t* test with exact *p* values indicated. Data are shown as mean ± S.D. (error bars) as well as with individual data points from each biological replicate.

Finally, we wanted to investigate whether functionally distinct populations of ATM *in vivo* have altered lipid compositions. CD9^+^ ATMs reside within crown-like structures in adipose tissue and display an M1-like phenotype, being proinflammatory and lipid-laden (13), while Ly6C^+^ ATMs reside outside of crown-like structures, display an M2-like phenotype, and are adipogenic (13). Therefore, we digested epididymal adipose tissue from high-fat-fed C57Bl6/J male mice, isolated CD9^+^ and Ly6C^+^ macrophage populations using fluorescence-activated cell sorting (FACS) and performed mass spectrometry lipidomics on the isolated cell populations. Principal component analysis (PCA) differentiated CD9^+^ and Ly6C^+^ macrophages, indicative of a difference in global lipid composition between the two cell types (Fig. 6*A*). Somewhat unexpectedly, we observed increased total TG levels in the M2-like Ly6C^+^ macrophages (Fig. 6*B*). Interestingly, there was a marked difference in the composition of the TG pool, with M1-like CD9^+^ macrophages having an increased proportion of PUFA-containing TG species, and Ly6C^+^ macrophages having an increased proportion of saturated and monounsaturated fatty acid–containing TG species (Fig. 6*C*). This finding is consistent with our data showing an increased proportion of PUFA-containing TG species in M1 macrophages (Figs. 1 and S1). We also noted significant differences in phospholipid abundance between CD9^+^ and Ly6C^+^ macrophages. Specifically, while the proportion of PC within the total PL pool was similar between cell types (Fig. 6*D*), CD9^+^ macrophages had higher levels of diacyl PC and lower levels of ether-PC relative to Ly6C^+^ macrophages (Fig. 6, *E* and *F*). Differences in PE composition and PS were also observed, while the proportion of PI was unaltered (Fig. 6, *G*–*K*). Finally, we also noted a marked difference in the proportions of major sphingolipid classes, ceramide, hexosyl ceramide, and sphingomyelin, between CD9^+^ and Ly6C^+^ macrophages (Fig. 6, *L*–*N*), indicative of differences in sphingolipid metabolism between cell types. These observations suggest that even though they are resident within the same adipose tissue depot, and have been fed the same HF diet, distinct ATM populations have alterations in their lipid metabolism.

## Discussion

Since the discovery of macrophage recruitment into adipose tissue in response to stimuli such as fasting (5), cold (32), and obesity (33), the interactions of macrophages with fatty acids have been intensely studied and linked to two distinct outcomes (1): the promotion of metabolic homeostasis, and (2) the deterioration of metabolic homeostasis as a result of the initiation of macrophage-intrinsic inflammatory processes (11). These contrasting responses are likely due to the considerable phenotypic heterogeneity displayed by tissue-resident macrophages (4). Indeed, macrophages recruited to adipose tissue in response to fasting or cold mediate adaptive responses and are typically M2-like macrophages (5, 32), while those present within the hypertrophied adipose tissue characteristic of obesity disrupt metabolic homeostasis and are typically M1-like inflammatory macrophages (9, 11). It is well established that numerous aspects of lipid metabolism are rewired during macrophage polarization (21) and we hypothesized that macrophage polarization state would influence the channeling of exogenous fatty acids into specific lipid biosynthetic pathways. Consistent with this idea, we show that exogenous fatty acids are preferentially channeled into TGs and CEs in M1 macrophages, while in M2 macrophages exogenous fatty acids are channeled toward GPL and sphingolipid synthesis.

Herein, we polarized macrophages into the M1 and M2 states with LPS + interferon-γ and interleukin-4, respectively. While this *in vitro* model of macrophage polarization is an oversimplification of the far more nuanced effector phenotypes present in tissue-resident macrophages *in vivo*, it nonetheless provides an important conceptual framework with which to understand macrophage biology. Indeed, recent studies using this *in vitro* model to study the phenotypes associated with distinct macrophage polarization states have revealed new biological insights into macrophage metabolism with important *in vivo* consequences (4, 34).

The observation herein that in the absence of exogenously supplied fatty acids M1 macrophages have higher levels of TGs and CEs is consistent with previous work demonstrating that activation of toll-like receptor (TLR) 4 by LPS induces TG and CE synthesis (27, 28). Several recent studies have begun to elucidate the importance of lipidome remodeling in general and increased TG and CE synthesis specifically, following TLR activation (28). In particular, lipid droplets, of which TG and CE are the principal constituents, were recently demonstrated to play important antibacterial roles (35). In addition, increased TG synthesis was demonstrated to be required for macrophage inflammatory functions, in particular the production of PGE_2_ (26). PUFAs such as AA are required for the production of eicosanoids such as PGE_2_ (36). In the present study, we observed that while M1 macrophages had higher levels of nearly all of the TG species we assessed, these differences were the most marked for PUFA-containing TG species. This increase in PUFA-containing TG species in M1 macrophages may serve as a substrate pool that is available for oxygenation reactions and the consequent production of oxygenated signaling lipids when required (26).

When not appropriately buffered, an increase in the concentrations of intracellular free fatty acids can lead to lipotoxicity. One of the principal mechanisms by which cells guard against free fatty acid–induced lipotoxicity is *via* the diacylglycerol acyltransferase (DGAT)-mediated esterification of excess free fatty acids into TGs (37, 38, 39). This process sequesters fatty acids, in the form of TGs, within lipid droplets, thereby preventing their utilization within lipid metabolic pathways that can induce lipotoxicity (40). M1 macrophage polarization increases *de novo* fatty acid synthesis and/or lipid uptake (28) and therefore, it is possible that the increase in TGs that accompanies M1 macrophage polarization may act to buffer elevated intracellular free fatty acid levels, thereby limiting their lipotoxic effects.

The major goal of the present study was to determine if macrophage polarization state affected the channeling of exogenous fatty acids into specific intracellular lipid pools. Our motivation behind this goal is that ATM subsets are differentially sensitive to the inflammatory effects of a high lipid environment. For example, macrophages recruited to the adipose tissue of mice that have been fasted or exposed to cold stress (4 °C), conditions that increase adipose tissue lipolysis and therefore fatty acid levels within the extracellular adipose tissue environment, are not inflammatory and instead express markers associated with M2 polarization (5, 32). Using single-cell RNA-seq, Lazar and colleagues have recently identified two distinct populations of macrophages within obese adipose tissue (1): a CD9^+^ population that colocalizes with crown-like structures, is lipid-laden, and expresses inflammatory molecules, and (2) an Ly6C^+^ population that is uniformly distributed throughout the adipose tissue, is not lipid-laden, and has a gene expression profile that would be expected to facilitate the maintenance of normal adipose tissue physiology (13). We hypothesized that one mechanism by which ATM subsets are protected from/sensitive to lipid-induced inflammation may relate to differences in the incorporation of exogenous fatty acids into specific endogenous lipid pools. Consistently, we observed that M1 macrophages accumulated substantially more TG and CE than M2 macrophages when exposed to exogenous fatty acids of varying chain length and saturation status. While an elevation in neutral lipids is associated with macrophage inflammation, TGs and CEs are themselves relatively biologically inert and are unlikely to drive inflammation directly (40). Instead, the increase in TGs in M1 macrophages following exposure to exogenous fatty acids is likely a protective response that limits the lipotoxic effects of elevated intracellular free fatty acids. Compared with M1 macrophages, TG accumulation in M2 macrophages exposed to exogenous fatty acids was markedly lower. One of the well-described differences in lipid metabolism between M1 and M2 macrophages is their fatty acid oxidation capacity, which is higher in M2 macrophages (23). This increased capacity to channel fatty acids toward oxidation may limit the accumulation of intracellular free fatty acids, thereby decreasing the need to esterify them into TGs.

An important finding of the present study was the observation that concomitant with their reduced esterification into TGs, exogenous fatty acids were incorporated into alternate lipid metabolic pathways in M2 macrophages. Specifically, while exogenous fatty acids increased the levels of numerous diacyl-phospholipids, ether and vinyl-ether phospholipids, and sphingolipids in both M1 and M2 macrophages, these increases were significantly greater in M2 macrophages. Palmitate is the preferred substrate for the rate-limiting enzyme, serine palmitoyl-CoA transferase, in the *de novo* sphingolipid synthesis pathway (31). Using stable-isotope-labeled palmitate, we were able to show markedly increased enrichment of d_3_-palmitate within ceramide sphingoid bases in M2 macrophages, demonstrating enhanced rates of *de novo* ceramide synthesis. Enrichment of d_3_-palmitate within the amide-linked acyl chain of several ceramide species was also higher in M2 macrophages, although to a less marked extent than observed for the sphingoid base. Interestingly, elevated intracellular ceramide levels mediate lipotoxicity and inflammation in a number of cellular contexts (41). However, the ablation of ceramide synthesis specifically within macrophages does not affect the activation of inflammatory signaling pathways in macrophages (42), obesity-induced metabolic dysfunction, or adipose tissue inflammation (43, 44), indicating that an increase in ceramide levels within macrophages does not promote inflammation. The mechanistic basis underlying increased *de novo* ceramide synthesis in M2 macrophages is unclear but is likely unrelated to transcriptional changes as no difference in the expression of *Sptlc1*, *Sptlc2*, and *Cers1*-*6*, key enzymes in the *de novo* ceramide synthesis pathway, between M1 and M2 macrophages was previously reported (17). These data highlight the importance of functional approaches to examine potential alterations in metabolic pathways.

Exogenous fatty acids were channeled into diacyl-GPL species in both M1 and M2 macrophages, and this was modestly higher in M2 macrophages. Strikingly, however, we observed a marked increase in the channeling of exogenous fatty acids into ether and vinyl-ether glycerophospholipid synthetic pathways in M2 macrophages. Owing to their specific structures, ether and particularly vinyl-ether lipids have distinct biochemical and biophysical properties when compared with their diacyl counterparts (29). Notably, the vinyl ether bond is proposed to have antioxidant properties, which may limit inflammation driven by heightened levels of reactive oxygen species (ROS) (29). Accordingly, an increased channeling of fatty acids into vinyl-ether synthesis in M2 macrophages may contribute to controlling cellular oxidant stress. Mechanistically, it has been shown that M2 macrophages express higher levels of *Far1*, the enzyme that reduces fatty acids to fatty alcohols in the first committed step of ether lipid synthesis, and *Gnpat*, the rate-limiting enzyme in ether lipid synthesis (17). Results from our stable-isotope labeling experiments demonstrate that both palmitate and oleate are reduced to their respective fatty alcohols to be incorporated at the Sn1 position of the glycerol backbone and that this effect is far more marked in M2 macrophages.

In the final part of this work, we assessed the lipid composition of functionally distinct CD9^+^ and Ly6C^+^ ATM. Firstly, we demonstrated that Ly6C^+^ has an increased proportion of TG relative to CD9^+^ macrophages. This finding was counter to our hypothesis, as we considered that the more M1-like CD9^+^ macrophages would have higher TG levels, consistent with what we observed *in vitro*. However, that we failed to observe this is perhaps not surprising given the markedly different scenario that the *in vitro* and *in vivo* conditions we studied present, *e.g.*, acute *in vitro* stimulation with a highly inflammatory stimuli *versus* chronically tissue-resident macrophages. Of note, we did observe a marked alteration in the composition of the TG pool that was consistent with both our own and other *in vitro* work (26). Specifically, the more M1-like CD9^+^ macrophages had a significantly greater proportion of PUFA-containing TG species, while the M2-like Ly6C^+^ macrophages had a significantly greater proportion of saturated and monounsaturated fatty acid–containing TG species. Our *in vitro* studies demonstrate a marked difference in how M1 and M2 macrophages metabolize exogenous fatty acids. Our *in vivo* work demonstrates that under relatively homeostatic conditions (*e.g.*, mice were not subjected to acute stimuli that would activate adipose tissue lipolysis and therefore increase the supply of exogenous fatty acids to tissue-resident macrophages) CD9^+^ and Ly6C^+^ macrophages have distinct lipid profiles, whether the metabolism of exogenous fatty acids is likewise altered between CD9^+^ and Ly6C^+^ macrophages will require future investigations, *e.g.*, comparing how the cellular lipid content of CD9^+^ and Ly6C^+^ macrophages differ in response to stimuli such as fasting or cold exposure.

In conclusion, our results add significant new knowledge with regard to how lipid metabolism is reprogrammed by macrophage polarization. In particular, we show that macrophage polarization state markedly alters the channeling of exogenous fatty acids into endogenous lipid pools. We propose that these differences, in addition to previously described alterations in lipid metabolism between M1 and M2 macrophages such as differences in fatty acid oxidation capacity, may contribute to the sensitivity, in the case of M1 macrophages, and the resistance, in the case of M2 macrophages, of ATM to lipotoxicity and inflammation.

## Experimental procedures

### Animals and procedures

*In vitro* experiments—In all experiments, bone-marrow-derived macrophages (BMDMs) were generated from wild-type, male C57Bl6/J mice aged 4 to 8 weeks old. Mice were bred and housed at the Alfred Research Alliance Animal Facility and were maintained on a 12:12 h light:dark cycle. All procedures complied with the national guidelines for the care and use of laboratory mice and were approved by an institutional animal ethics committee (AMREP AEC). *In vivo* experiments—11-week-old C57Bl6/J mice were placed on a HFD (SF04-001, 43% energy from lipids; Speciality Feeds, Glen Forrest) for 10 weeks. After 10 weeks of HFD, mice were humanely culled and the epididymal adipose tissue collected; mice were not fasted prior to being culled. These procedures complied with the national guidelines for the care and use of laboratory mice and were approved by the institutional animal ethics committee of the University of Melbourne.

### Generation of M1 and M2 BMDM and treatment with nonlabeled fatty acids

Following CO_2_ asphyxiation, femurs and tibias were removed, cleaned, and bone marrow (BM) flushed from the bones with RPMI-1640 media containing GLUTAMAX (RPMI; Life Technologies). BM was centrifuged (500*g*, 5 min) and resuspended in RPMI media containing 5% Fetal Bovine Serum (FBS; Life Technologies) before cells were counted on an automated analyzer (Sysmex XS-1000i). BM cells (1 million/ml) were cultured in macrophage differentiation media (RPMI containing 10% FBS, 1% penicillin/streptomycin (Life Technologies), and 20 ng/ml macrophage-colony stimulating factor (M-CSF; PeproTech)) in a T75 flask. Following an overnight incubation (37 °C, 5% CO_2_), nonadherent cells were plated in 24-well tissue culture plates (Jet-Biofil) at a density of ∼4 × 10^5^ cells per well in 1 ml of macrophage differentiation media. After 3 days, an additional 1 ml of macrophage differentiation media was added. BM cells were considered to be fully differentiated into macrophages following 7 days of culture. To induce polarization to the M1 and M2 states, BMDMs were treated with 100 ng/ml ultrapure lipopolysaccharide (LPS; InVivogen) and 20 ng/ml interferon-y (IFNy; PeproTech) for M1 polarization, or 20 ng/ml IL-4 (PeproTech) for M2 polarization, in macrophage differentiation media for 24 h. *De novo* lipogenesis was inhibited by pretreating M0 (nonpolarized) macrophages with either an inhibitor of acetyl-CoA carboxylase (TOFA; 20 μM; Enzo Life Sciences) or the mitochondrial pyruvate carrier (UK5099; SigmaAldrich; 50 μM) for 1 h. M0 BMDMs were subsequently polarized to either the M1 or M2 states or remained as M0 macrophages. TOFA and UK5099 were present in the media during the 24 h M1 polarization. Unlabeled FAs (palmitic acid, P0500; oleic acid, O1008; arachidonic acid, 10931; docosahexaenoic acid, D2534; all from Sigma-Aldrich) were initially solubilized in ethanol before being added to filtered (0.2 μM Minisart filter; Sigma-Aldrich) RPMI media containing 5% FBS and 2% w/v bovine serum albumin (BSA; Sigma-Aldrich #A6003; used to complex the fatty acids) to give a final concentration of 200 μM. For experiments using stable-isotope labeled fatty acids, d_9_-oleate (Avanti Polar Lipids # 861809) and d_3_-palmitate (Sigma-Aldrich #615951), deuterated FAs were likewise initially solubilized in ethanol before being added to RPMI media containing 5% FBS and 0.2% w/v BSA to give a final concentration of 20 μM. FA mixtures were gently rocked for an hour at 37 °C to aid conjugation of the fatty acids to BSA. Once prepared, FAs or vehicle controls (BSA + ethanol) were added to polarized BMDM, after which BMDMs were washed (×2) with cold PBS and cells stored at −80 °C until further processing (Lipidomics and Western blotting).

### Lipidomics

#### Lipid extraction

BMDMs were scraped, collected in 200 μl of ice-cold PBS, sonicated (S-4000; Misonix), and protein concentrations determined (Thermo Fisher Scientific, #23225) to allow for data normalization during analysis. Samples were then dried overnight in a Speedvac (Thermo Scientific) in preparation for lipid extraction. Lipids were extracted using a single-phase chloroform/methanol extraction as described previously with modification for cultured cells (45, 46).

#### Targeted lipidomics analysis

Liquid chromatography–tandem mass spectrometry (LC-MS/MS) was performed according to previously published methods, with slight modification for cultured cells (45, 46, 47). Cellular extracts were analyzed using either (i) a 4000 Qtrap mass spectrometer (AB Sciex) with an Agilent 1290 series HPLC and a ZORBAX eclipse plus C18 column (2.1 × 100 mm 1.8 μm, Agilent) with the thermostat set at 60 °C to analyze the cell extracts; or (ii) an Agilent 6490 QQQ mass spectrometer with an Agilent 1290 series HPLC system and a ZORBAX eclipse plus C18 column (2.1 × 100 mm 1.8 μm, Agilent) with the thermostat set at 45 °C. Mass spectrometry analysis was performed using dynamic scheduled multiple reaction monitoring (MRM) in positive ion mode; transitions, internal standards, and conditions have been previously reported (47). Lipids were identified based on their retention time, precursor, and product ions. For the experiments using nonlabeled fatty acids, data was analyzed in MultiQuant 2.1.1 (AB Sciex) software. Lipid abundances were determined by normalizing the area under the chromatogram for each lipid species against the corresponding internal standard. Lipid abundance was normalized to the total PC of the respective sample. Total PCs were not different between groups.

#### Tracer lipidomics

Cells were treated with either BSA, d_9_-oleate, or d_3_-palmitate for the durations indicated within specific figure legends and were subsequently extracted as described above. Mass spectrometry transitions were modified to detect trace incorporation of deuterated fatty acids into lipids in a targeted manner based on lipid species of interest. Lipid species expected to incorporate d9-oleate had their precursor mass offset by 9 Da, while the product ions were offset depending on lipid class fragmentation patterns. In instances where double incorporation was possible, the precursor mass was also offset by 18 Da. This was additionally performed for d3-palmitate but with 3 and 6 Da for single and double incorporation, respectively. The corresponding nonlabeled species were also measured. Data was analyzed using Agilent MassHunter 10.0. Percent enrichment was calculated as: Area of the labeled species/(Area of labeled species + Area of non-labeled species) ×100.

### Western blot analysis

To confirm that BMDMs were effectively polarized into the M1 and M2 states, BMDMs were analyzed by Western blot. Briefly, after being washed (×2) with ice cold PBS, 100 μl of 2× Laemmli buffer containing 200 μM dithiothreitol was directly added to wells, and cells were scraped and collected before being incubated at 95 °C for 10 min. Samples were resolved by SDS polyacrylamide gel electrophoresis and then transferred to 0.2 μm nitrocellulose membranes (Bio-Rad) by wet transfer at 4 °C. Membranes were washed with Tris-buffered saline containing Tween 20 (TBST) for ∼30 min before being blocked in TBST containing 5% skim milk powder for 1 h, and finally washed in TBST, all conducted at room temperature. Membranes were incubated overnight at 4 °C on a rotary shaker with antibodies (All from Cell Signalling Technology – iNOS, #13120; Arginase-1, #93668; GAPDH, #2118; HSP90, #4877; all at 1:1000 dilution) made up in TBST containing 2.5% (wt/vol) BSA and 0.01% (wt/vol) NaN_3_. Membranes were then washed (TBST) for 30 min, incubated with secondary antibody (Anti-rabbit IgG (H + L)-HRP Conjugate, Bio-Rad #1706515; 1:2000 in TBST +2.5% BSA) for 1 h at room temperature, and then washed (TBST) for a further 30 min before being visualized using a chemiluminescent substrate (mixture of Thermo Fisher Scientific chemiluminescent substrates – #34095 and #34577) on a ChemiDoc XRS system (Bio-Rad). Where required, membranes were stripped (Thermo Fisher Scientific, #21059) to allow for reprobing with a different primary antibody.

### Fatty acid uptake assessment using copper-catalyzed azide-alkyne chemistry

Copper-catalyzed azide-alkyne cycloaddition (“click”) chemistry-based approaches have been used to visualize fatty acids *in vitro* and *in vivo* (48). Using commercially available reagents, we developed a click-chemistry-based method for assessing fatty acid uptake by flow cytometry. This method has the advantage over fluorescently labeled fatty acids (*e.g.*, BODIPY-labeled fatty acids) in that the fatty acid used is very minimally modified (*i.e.*, an alkyne handle replaces the terminal methyl group). Posttreatment, alkyne-FAs can then be visualized using reporter-tagged azide-conjugates, *e.g.*, azide-AlexaFluor488. Studies to date have shown that alkyne fatty acids are metabolized in a similar manner to fatty acids, making them more physiologically representative of their native counterparts compared with fatty acids directly conjugated to a fluorophore.

Alkyne-palmitate (Cayman Chemical, #13266) and alkyne-oleate (Cayman Chemical, #9002078) were initially solubilized in absolute ethanol and then added to RPMI containing 2% FBS and 0.2% w/v BSA to attain the final desired concentrations (shown in Fig. S2). Mixtures were gently rocked for 30 min at 37 °C to aid conjugation. Next, polarized BMDMs were treated with alkyne-FA mixtures for 10 min before being washed (×2) with PBS, treated with Accutase (Sigma-Aldrich, #A6964) for 15 min at 37 °C, 5% CO_2_. After which, sterile PBS supplemented with 10% FBS was added and any remaining adherent cells were dislodged by gentle, repeated pipetting before being collected in Eppendorf tubes, and centrifuged (500*g* for 5 min; 4 °C). Next, cells were resuspended in Fixation and Permeabilization solution (BD Cytofix/Cytoplasm, #554722) and incubated for 20 min on ice. The process was stopped with Perm/Wash buffer (BD Phosflow, #557885), and cells were spun (500*g* for 5 min; 4 °C) and then washed (FACS buffer; 500 μl). Next, a cocktail of Click-iT reaction reagents (which included 5 μM azide-conjugated AlexaFluor488; LifeTechnologies, #A10266) was prepared according to the manufacturer’s protocol (Click-iT Cell reaction buffer kit, Thermo Fisher Scientific, #C10269) and added to cells (30 min at room temperature, in the dark). Controls that were treated identically except for the absence of CuSO_4_ in the Click-iT cocktail, and which are therefore unable to undergo the click reaction, were included to account for background fluorescence. FACS buffer was then added to wash the cells, samples were spun (500*g* for 5 min; 4 °C) and then resuspended in an antibody cocktail containing macrophage-specific markers (Anti-F4/80-PE, Biolegend #123110, 1:200; Anti-CD11b-PE-Cy7, Biolegend #101216, 1:200) and a viability dye (Ghost dye-BV510, Tonbo #10931, 1:1000) and incubated on ice, in the dark, for 30 min. Cells were washed and transferred to FACS tubes for analysis using an LSRII Fortessa (BD Biosciences) flow cytometer with FACSDiva software. The gating strategy used to identify macrophages and to quantify the mean fluorescence intensity of the alkyne-FA/azide-AlexaFluor488 conjugate is shown in Figure S2.

### Isolation of adipose tissue macrophages

Adipose tissue was placed in a 5 ml tube with 2 ml liberase (0.03 mg/ml in RPMI; SigmaAldrich) and finely minced. Minced adipose tissue was moved to a small Petri dish and then incubated in a rotary shaker for 45 min at 37 °C. Next, 10 ml of cold RPMI was added to the Petri dish, and this RPMI/minced adipose tissue mixture subsequently filtered (250 μm) and transferred to a 15 ml falcon tube. Samples were then spun (3000 rpm, 10 min, 4 °C), the floating adipocytes and supernatant removed, 1 ml of red blood cell lysis buffer added for 5 min, the reaction stopped with the addition of ∼5 ml of FACS buffer, and samples spun again (3000 rpm, 5 min, 4 °C). After removing the supernatant, samples were resuspended in 1 ml FACS buffer and cells counted (Sysmex XS-1000i). Cells were then incubated with the following antibodies (1:400 dilution): CD45-BUV395 (Cat# 564279; BD Biosciences), Ly6C-PerCP (Cat# 45-5932-82), CD11b-APC (Cat# 17-0112-81; Thermo Fisher Scientific), CD64-BV650 (Cat# 740622; BD Biosciences), F4/80-FITC (Cat# 123108; Biolegend), CD9-PE (Cat# 12-0091-83; Thermo Fisher Scientific), and a viability dye (Ghost Dye BV510; TONBO biosciences) for 30 min, on ice, in the dark. After 30 min, ∼1 ml of FACS buffer was added, cells spun (2800 rpm for 3 min), and the cell pellet resuspended in ∼300 μl of FACS buffer and filtered (40 μm) prior to sorting on a BD FACSAria Fusion flow cytometer at the AMREP flow cytometry core facility (Burnet Institute). CD9^+^ and Ly6C^+^ cells were gated essentially as described previously (13): for CD9^+^ cells—viable (Ghost Dye negative), single cells were initially gated, from which CD45^+^ cells were gated, from which CD11b^+^ Ly6C^−^ were gated, from which F4/80^+^ CD64^+^ cell were gated, from which CD9^+^ cells were gated for sorting; for Ly6C cells—viable (Ghost Dye negative), single cells were initially gated, from which CD45^+^ cells were gated, from which CD9^−^ cells were gated, from which CD11b^+^ Ly6C^+^ cells were gated for sorting. We were able to sort ∼70,000 to 250,000 CD9^+^ macrophages and ∼5000 to 60,000 Ly6C^+^ macrophages. After cells were sorted, they were washed once with PBS, and the cell pellets stored at −80 °C in 100 μl PBS. Prior to lipidomic analysis, samples were lyophilized in a Savant SpeedVac (Thermo Scientific) and subsequently reconstituted in milliQ water. In this experiment, samples were extracted using the butanol:methanol method, as we have described previously (49), and processed as described in the targeted lipid analysis section. To investigate the compositional differences between CD9^+^ and Ly6C^+^ macrophage populations, we utilized two lipid normalization approaches, (1) to investigate TG compositional changes, we normalized each TG species to the sum total triglyceride concentration, (2) to investigate phospholipid changes between cells types, we examined each lipid class total relative to the total glycerophospholipid pool. This was done similarly for each sphingolipid class and the total sphingolipid pool. While we present some of the data from this work in Figure 6, we have created a supplemental excel file (File S1) that contains data on all the lipid species we have measured.

### Statistical analysis

Data were analyzed by unpaired *t* test, one-way analysis of variance (ANOVA), or two-way ANOVA; the specific statistical tests used are indicated in the figure legends. PCA was performed in R (v4.0.5) using the FactoMineR and factoextra packages. Data was log-transformed prior to PCA. In the TG species heatmap in Figure 6*C*, the TG species shown are those that were significantly different (unpaired *t* test) after performing a Benjamini–Hochberg false discovery rate correction set at 10%. One biological replicate in the M1 BSA group (from the experiment in which the four different FAs were used) was excluded in all analysis as it was a clear technical artefact. In the *in vivo* experiment, two mice were excluded from the final analysis due to low cell numbers obtained from the sort (<2000 Ly6C^+^ cells). A *p*-value of less than 0.05 was used to define statistical significance, although more precise *p*-values are indicated in the figures. All data are shown as the mean ± S.D.

## Data availability

All data are contained within the manuscript and supporting information or are available from the authors: Graeme I. Lancaster (Graeme.lancaster@baker.edu.au) and Andrew J. Murphy (Andrew.murphy@baker.edu.au) upon request.

## Supporting information

This article contains supporting information.

## Conflict of interest

The authors declare that they have no conflicts of interest with the contents of this article.
