# Supplementary material for: Macrophage polarization state affects lipid composition and the channeling of exogenous fatty acids into endogenous lipid pools
Source: J Biol Chem. 2021 Oct 23;297(6):101341. doi: 10.1016/j.jbc.2021.101341 (PMC8604758; doi:10.1016/j.jbc.2021.101341)
Supplement: Figures S1–S5 [file mmc1.pdf]

Macrophage polarization state affects lipid composition and the channelling of exogenous fatty acids into endogenous lipid pools

Pooranee K. Morgan[1,2,5], Kevin Huynh[1,5], Gerard Pernes[1,5], Paula M. Miotto[3], Natalie A. Mellett[1], Corey Giles[1], Peter J. Meikle[1], Andrew J. Murphy[1,4,6] and Graeme I. Lancaster[1,4,6]

Running Title: Macrophage polarization state and lipid metabolism

Keywords: macrophage, fatty acid metabolism, phospholipid metabolism, sphingolipid, cell metabolism

[1] Baker Heart and Diabetes Institute, 75 Commercial Road, Melbourne, Australia, 3004.

[2] School of Life Sciences, La Trobe University, Melbourne, Australia, 3086.

[3] Department of Anatomy and Physiology, School of Biomedical Sciences, University of Melboure, Melbourne, Australia, 3010.

[4] Department of Immunology, Monash University, Melbourne, Australia, 3004.

[5] These authors contributed equally to this work.

[6] Co-address for correspondence

Supplementary Figure 1

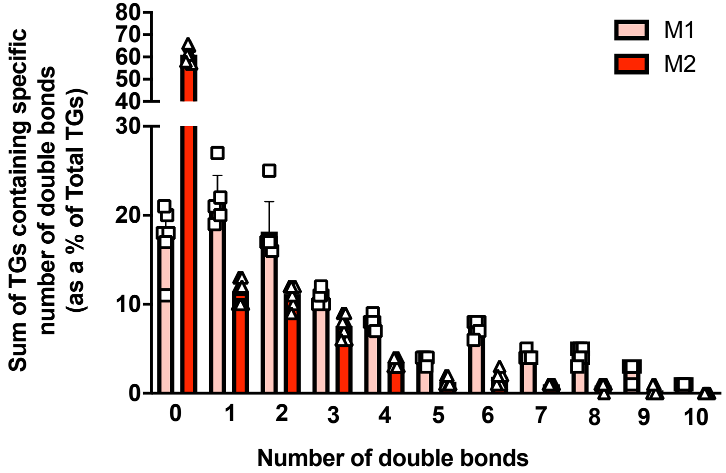

Supplementary Figure 1. The composition of the TG pool is different in M1 and M2 macrophages. TG composition was assessed in M1 and M2 BMDM by expressing the sum all of the individual SIM TG species of a given saturation status as a % of total TG. Data are shown as mean ± S.D. (error bars) as well individual data points from each biological replicate.

Supplementary Figure 2

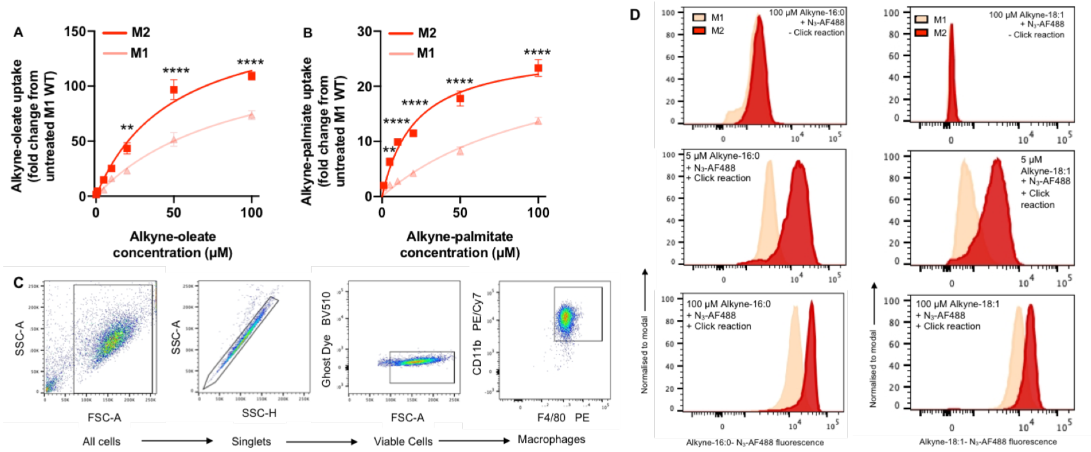

Supplementary Figure 2. Fatty acid uptake is higher in M2 relative to M1 macrophages. *A* and *B*, Alkyne fatty acid uptake was assessed in M1 and M2 BMDM following 10 min of treatment with either alkyne-palmitate (16:0) or alkyne-oleate (18:1) at the indicated doses. *C* and *D*, Illustrate the gating strategy used to define viable macrophages following treatment (*C*) and provide representative histograms of the typical fluorescence changes observed following treatment with the alkyne fatty acids in the presence and absence of $CuSO_4$, which is required to catalyze the click reaction that initiates azide/alkyne conjugation (*D*). 2-way ANOVA with Tukey's HSD test was used to determine statistically significant differences. ** and **** indicate significant differences between pairwise comparisons of M1 *vs*. M2 at individual alkyne fatty acid doses at the following significance level: p<0.01 and p<0.00001, respectively. N=3 biological replicates in all groups. Data are shown as mean ± S.D. (error bars).

Supplementary Figure 3

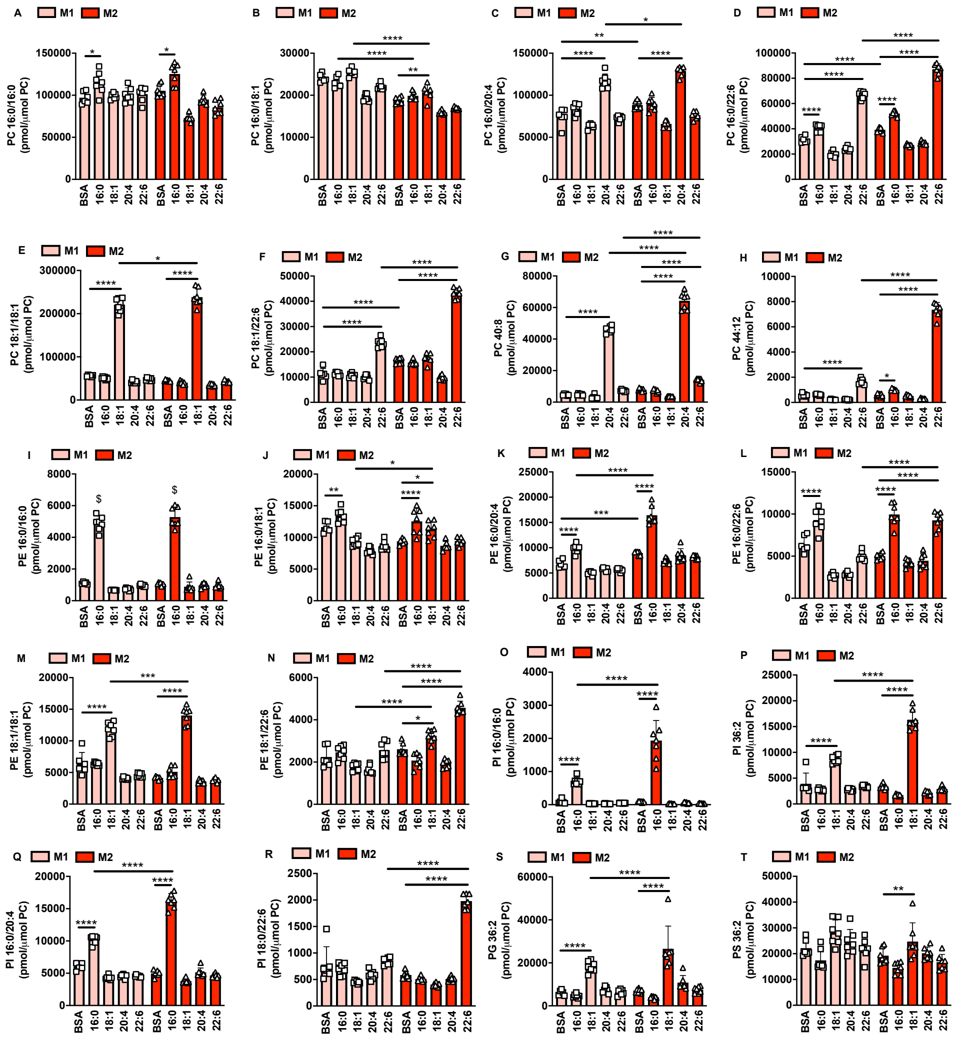

Supplementary Figure 3. Exogenous fatty acids increase glycerophospholipid levels to a greater extent in M2 relative to M1 macrophages. M1 and M2 BMDM were treated individually for 4 hrs with 200 µM palmitic acid (16:0), oleic acid (18:1), arachidonic acid (20:4), docosahexaenoic acid (22:6), or vehicle control (BSA). Samples were analysed by MS lipidomics and the levels of individual phosphatidylcholine (PC) (*A-H*), phosphatidylethanolamine (PE) (*I-N*), phosphatidylinositol (PI) (*O-R*), phosphatidylglycerol (PG) (*S*), and phosphatidylserine (*T*) glycerophospholipid species were determined. 2-way ANOVA with Tukey's HSD test was used to determine statistically significant differences. *, **, ***, and **** indicate significant differences between pairwise comparisons of the indicated groups at the following significance level: p<0.05, p<0.01, p<0.001, and p<0.00001, respectively. $$$$ indicates a significant difference (main effect) between 16:0 and BSA treated BMDM at the following significance level: p<0.00001. Data are shown as mean ± S.D. (error bars) as well as with individual data points from each biological replicate.

Supplementary Figure 4

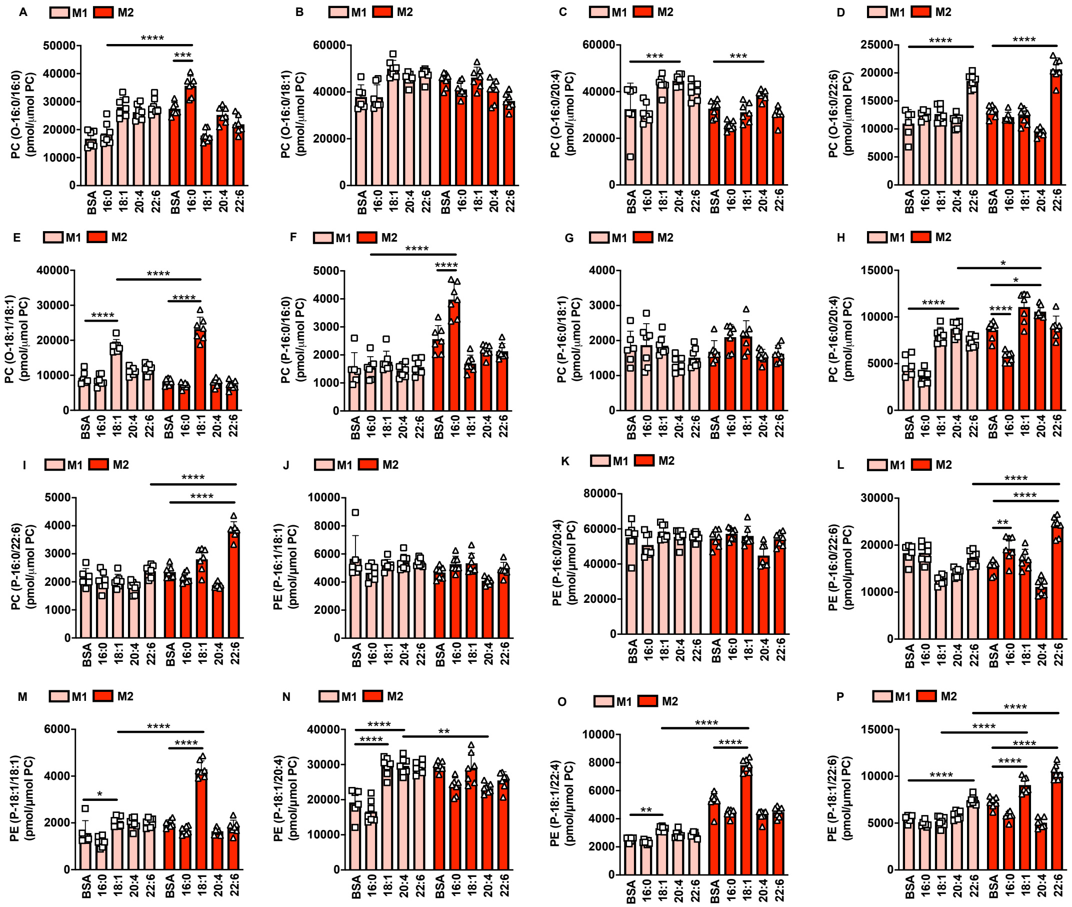

Supplementary Figure 4. Exogenous fatty acids increase ether and vinyl-ether glycerophospholipid levels to a greater extent in M2 relative to M1 macrophages. M1 and M2 BMDM were treated individually for 4 hrs with 200 µM palmitic acid (16:0), oleic acid (18:1), arachidonic acid (20:4), docosahexaenoic acid (22:6), or vehicle control (BSA). Samples were analysed by MS lipidomics and the levels of individual ether PC (*A*-*E*), vinyl-ether PC (*F*-*I*), and vinyl-ether PE (*J*-*P*). were determined. 2-way ANOVA with Tukey's HSD test was used to determine statistically significant differences. *, **, ***, and **** indicate significant differences between pairwise comparisons of the indicated groups at the following significance level: $p<0.05$, $p<0.01$, $p<0.001$, and $p<0.00001$, respectively. Data are shown as mean ± S.D. (error bars) as well as with individual data points from each biological replicate.

Supplementary Figure 5

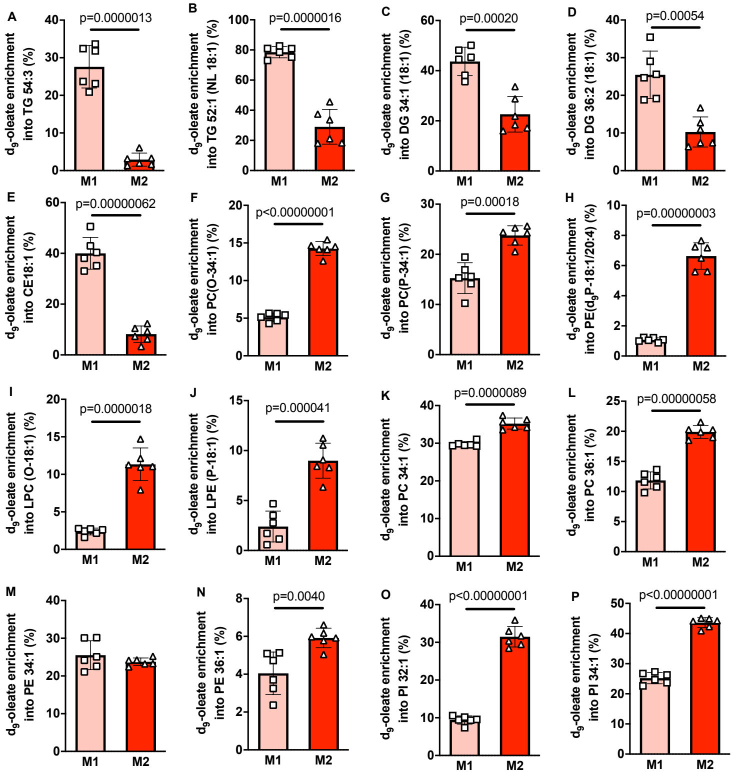

Supplementary Figure 5. Deuterated (d_9)-oleic acid is enriched within neutral lipids in M1 macrophages and within glycerophospholipids within M2 macrophages. M1 and M2 BMDM were treated with d_9-oleic acid for 4 hrs and enrichment within individual TG (*A*-*B*), DG (*C* and *D*), CE (*E*), ether PC (*F*), vinyl-ether PC and PE (*G*-*H*), ether lyso (L)PC (*I*), vinyl-ether LPE (*J*), PC (*K* and *L*), PE (*M* and *N*), and PI (*O* and *P*) species was determined by MS lipidomics. Data was analysed by unpaired t-test with exact p values indicated. Data are shown as mean ± S.D. (error bars) as well as with individual data points from each biological replicate.
